# Clinical Memoranda from the Sisters’ of Charity Hospital1Read before the Surgical Section of the Buffalo Academy of Medicine, Tuesday evening, June 5, 1894.

**Published:** 1894-07

**Authors:** Herman Mynter

**Affiliations:** Professor of Surgery, Niagara University, Buffalo, N. Y.


### CLINICAL MEMORANDA FROM THE SISTERS' OF CHARITY HOSPITAL.[1]

BY HERMAN MYNTER, M. D.,
Professor of Surgery, Niagara University, Buffalo, N. Y.

### CHOLECYST-DUODENOSTOMY AND GASTRO-ENTEROSTOMIES BY AID OF MURPHY'S BUTTON.

WHILE operations on the gall-bladder have been performed as long ago as in the middle of last century, it is first during the last ten or fifteen years that improved methods have been devised, by aid of which a moderately favorable prognosis could be obtained. And yet the progress has been so rapid, that operations, which but a few years ago were universally employed, are obsolete now or will soon be considered so.

We owe this progress, as so many other advances in surgery, to American surgeons ; in this case to Dr. John B. Murphy, of Chicago, whose anastomosis button, by its simplicity and mechanical perfection, and the ease and celerity with which it may be applied, seems to fulfil all indications for a safe and reliable removal of gallstones from the gall-bladder itself.

The question arises, When ought we to operate in cases of jaundice ? It is impossible to give a distinct diagnosis in each case. Jaundice may be the result of gallstone impaction in the common duct, or of cancer of the pancreas or liver, involving the duct, or simply of a gastro-duodenal catarrh. In many cases, of course, the symptoms will be sufficiently prominent to give a differential diagnosis ; in others the diagnosis will be obscure, unless we open the abdomen.

---

1. Read before the Surgical Section of the Buffalo Academy of Medicine, Tuesday evening, June 5, 1894.

I need scarcely mention that an impacted gallstone, if sufficiently large, in the common duct gives jaundice, and, secondarily, swelling of the gall-bladder, while an impaction in the cystic duct is not followed by jaundice, and may not be followed by swelling of the gall-bladder, unless cholecystitis supervenes. On the other hand, swelling of the gall-bladder, with or without jaundice, may be the result of a carcinoma of the gall-bladder itself, or neoplasms in the pancreas and liver, involving the common duct, or of an empyema, the result of a cholecystitis with or without gallstones.

When ought we, therefore, to discard medical treatment, and have recourse to the only means which can clear up the diagnosis —laparatomy ?

I believe that jaundice ought to be treated by laparatomy after a course of medical treatment of several weeks has demonstrated the improbability of relieving the trouble by this means.

The abdomen having been opened by a vertical incision at the outer edge of the rectus muscle, we may examine the gall-bladder for stones in the common, cystic or hepatic ducts, and if found, particularly in the gall-bladder, remove them with scarcely any danger to the patient. If carcinoma of the gall-bladder should be found, it is entirely feasible to remove the gall-bladder by cholecystectomy. If the common duct should be found obstructed by a neoplasm, we may, at least, relieve the jaundice by a cholecystenterostomy, without any more danger to the patient than accompanies an explorative antiseptic laparatomy, and that is *nihil.*

The older operations for gallstones consisted in: (1) suture of the gall-bladder to the parietal peritoneum with secondary incision, *i. e.*, cholecystotomy in two sittings; (2) suture with immediate incision, *i. e.*, cholecystotomy in one sitting; (3) incision of gall-bladder followed by immediate suture and reposition in abdominal cavity, *i. e.*, ideal cholecystotomy. Of these three operations, the first has given the most favorable results, the mortality being ten per cent., six deaths in fifty-nine cases.

The second operation has given a mortality of 19 per cent. in 201 cases, but both the first and second operations have the great disadvantage that a biliary fistula is left in a large number of cases, estimated at 31 per cent., so that perfect recovery has only been obtained by these operations in about 50 per cent. The third operation, ideal cholecystotomy, has given a mortality of 23 per cent. Compare the results of these operations with the most modern operation, scarcely as yet known to physicians, the cholecyst-enterostomy, or cholecyst-duodenostomy, by aid of Murphy's button, with a mortality of *nihil* and a complete recovery of 100 per cent. in seventeen cases, or if I add one of my own, eighteen cases, and you will probably agree with me, that the problem of removing gallstones by operation has been most ably and brilliantly solved by this new device.

The operation is performed in the following way: after the abdomen has been opened and the gall-bladder isolated and drawn out of the wound, a running thread is inserted around a line one-third longer than the incision to be made, and going through all the layers of the organ. The incision is thereafter made and the gallstones removed. It is not necessary to remove all the gallstones, as they will pass away after the button has been passed. One-half of the button is now inserted with a forceps and the running thread tied around the cup. A similar thread and incision is made in the duodenum, opposite the mesentery and below the head of the pancreas, the button inserted and the two halves firmly pressed together. The spring in one of the cups maintains pressure till the button sloughs off, and it is voided by rectum in from seven to twenty or twenty-five days. The gall-bladder shrinks thereafter, forming a canal of the size of the common duct. This operation is, according to Murphy, indicated: (1) in all cases in which it is desired to drain the gall-bladder; (2) in all cases of cholelithiasis with obstruction of the common duct; (3) in all cases of cholecystitis, with or without gallstones; (4) in all profusely discharging biliary fistulas. It is contra-indicated: (1) when the gall-bladder is too small for insertion of the button; (2) where adhesions are so extensive that the bladder and duodenum cannot be approximated; (3) where the ductus cysticus is obliterated, in which cases cholecystectomy is indicated.

When a stone is impacted in the common duct, attempts have occasionally been made of removing it either by crushing it through the walls with instruments protected by a rubber covering, or by dividing it by a needle introduced through the wall, or, lastly, by incising the duct, removing the stone and closing the wound by suture. This last operation is very difficult, on account of the deep position of the duct, and the mortality is about forty per cent.

The Murphy button may be used to advantage on other organs than the gall-bladder. In a recent case, operated seven weeks ago, I made an anastomosis between the stomach and the duodenum, on account of cancerous stricture of the pylorus. The case was entirely successful, all vomiting stopped, and the patient left the hospital in three weeks greatly improved, able to eat and retain his food. His life will, probably, be lengthened a good many months by this operation. In another case of cancer of the pylorus, in which the patient was extremely exhausted from starvation from vomiting, death occurred twelve hours after the operation. The operation was performed in less than fifteen minutes, and she died simply from exhaustion. In a third case of cancer of pylorus, in which the patient was in a state of extreme inanition, I made a gastro-duodenostomy a few weeks ago, using the smallest of Murphy's buttons, as the others of my set were all in use. For seven days everything went well, vomiting had ceased, and the patient was improving and feeling well, when she suddenly complained of severe burning pain in the abdomen, as if "melted lead was being poured down among the bowels." She collapsed and died in two hours. The *post-mortem* showed that the button had slipped out of the stomach, leaving a large opening, through which the contents had entered the abdominal cavity. The small diameter of the button necessarily allowed only a small brim of the hypertrophied wall of the stomach to be compressed between the cups, and the accident occurred partly on this account, partly from muscular contractions of the stomach. I consider it of the utmost importance to publish this case, and shall, in all future gastro-enterostomies, use a running suture around the button as a means of safety. I do not believe it could have occurred in a cholecyst-enterostomy, where the muscular force, probably, is lacking.

I question whether a gastro-duodenostomy may not be the proper thing to do in recurrent ulcers of the duodenum. We do not, of course, know in a given case whether the ulcer is duodenal or gastric, but both result in stenosis with consecutive dilatation of the stomach, hypertrophy and, later, atrophy of the wall.

Dr. Carle, of Turin, in the last international congress, at Rome, reported fourteen operations on the stomach for non-malignant affections, of which only one died. Eleven were pyloro-plastic operations, one a gastro-enterostomy for stenosis with hemorrhages, one a divulsion and one a resection of the pylorus. His conclusions are: (1) In rebellious chronic gastric catarrh it is permissible to resort to exploratory laparatomy. (2) When a condition is found to exist rendering evacuation of the stomach difficult or impossible, it is necessary to perform some operation to remedy the defect.

(3) It is absolutely essential to provide for free evacuation of the stomach, for it is better that the organs should be incontinent than that stagnation of the gastric contents should exist. (4) The successful result in the case of gastro-enterostomy proved to his satisfaction that ulcer with hemorrhage can be treated in this way more satisfactorily than by resection.

Mikulicz's pyloro-plastic operation is difficult, and takes a long time, probably several hours, to perform. Loreta's operation of divulsion has the same disadvantage as divulsion in strictures,—that there is nothing to prevent a return of the constriction. With Murphy's button, on the other hand, we can establish a new communication in a very short time (about ten minutes), without danger of shock from long exposure of the abdominal organs, and I should, therefore, consider this method preferable. In cases of ulcer with hematemesis and stenosis we may expect, it seems to me, the ulcer to heal and form a firm cicatrix, as it will not be continually irritated by the passage of food and fermentation.

Lastly, I wish to report two cases of cholecyst-enterostomy :

Case XVII.—Mr. M., aged 24, entered the Sisters' hospital in March, 1894, with the following history: He had been taken sick fourteen months previously with a deep-seated, heavy, dull pain in the epigastrium, followed, at irregular intervals, by sharp, crampy pains, beginning in the same region and extending all over the abdomen. Jaundice occurred shortly after, and had continued since in increasing degree, so that he was now of a deep yellow, or rather greenish, color. He had continually had clay-colored stools, dark urine, intense itching, nose-bleeding. He had complained of increasing emaciation and weakness, and I do not remember to have seen a more utterly prostrated and cadaverous-looking patient. The liver was enlarged, extending three inches below the lower border of the ribs. A rounded tender swelling was felt to the right of the median line in the epigastrium.

That we here had a case of stenosis of the common duct admitted of no doubt, but the cause was unknown, although the probable cause, judging from the history, might seem to be gallstones. Laparatomy was, therefore, performed along the right border of the rectus muscle. An enormously dilated gall-bladder, as large as the head of a child, was seen, strongly adherent to transverse colon. The adhesions were loosened and the gall-bladder drawn out through the wound and punctured. It contained about a pint of thin, yellow-greenish fluid mixed with mucus. No stones could be felt, but a deep hard tumor was felt behind, probably in the pancreas. A cholecyst-duodenostomy was thereafter made with Murphy's button, No. 2, and the wound closed.

He lived six days after the operation, and died of prostration without peritonitis. On the second day the stools commenced to become brownish in color, the urine cleared up considerably, and he complained only of extreme weakness.

*Post-mortem* showed cancer of pancreas infiltrating and closing the common duct, and disseminated cancerous foci in the liver.

Case XVIII.—Mrs. K., sixty years of age, had for nine years suffered from periodical pain in the region of the liver, followed by severe vomiting, and treated for neuralgia of the stomach. She had never suffered from icterus. In February, 1894, she was taken sick with what was supposed to be an attack of appendicitis, had pain, vomiting, fever and a tender painful swelling in the right ileo-cecal region. She recovered, but had another attack in the beginning of April, 1894, which also was treated for appendicitis.

I was called to see her on April 14th, as she continued to have pain in the ileo-cecal region. There was felt here a deep-seated, oblong, movable and painful tumor, extending from McBurney's line downward and inward. The tumor was about three inches in length and, apparently, a couple of inches broad, and could be indistinctly followed upward toward the liver. An exploratory incision was made, on April 17th, by the usual oblique incision for appendicitis, and the tumor proved to be an enlarged, thickened and elongated gall-bladder, full of pus and gallstones, and adherent to colon ascendens and transversum. The usual suture was applied and the gall-bladder opened, and 126 gallstones removed, of different sizes, the largest as large as a hickory nut. Duodenum was found with difficulty and only by following the ileum upwards from the ileo-cecal valve. The button was thereafter inserted and the abdomen closed. The furtber course was favorable, and she left the hospital on the fourteenth day, stating that she had not felt so well for many years. The button could then still be felt beneath the scar. On the twenty-second day the button could not be felt any more, and she passed it on the twenty-third day. She has since felt in perfect health.

